# Pneumonias – a dynamic relationship between the human organism and the etiologic agent

**Published:** 2014-06-25

**Authors:** R Chiotan, M Chiotan

**Affiliations:** * "Prof. Dr. Marius Nasta" Institute for Respiratory Medicine, Bucharest; ** "Prof. Dr. Matei Bals" National Institute for Infectious Diseases, Bucharest

**Keywords:** pneumonias, defence mechanisms, infections, infectious agents, inflammation

## Abstract

Abstract

Pneumonias are usually acute infectious processes of the pulmonary parenchyma, being the result of the overcome of the natural defence mechanisms of the human organism, which lead to the penetration, persistence and multiplication of a biologic agent (which has become a pathogen agent) within the lungs. This is a phenomena which generates local manifestations (inflammation) and systemic manifestations, their variable dimension (which is conditioned by the state of the host and/or the virulence of the pathogen agent) explaining the clinical, paraclinical and biological dimensions of the phenomena. The current material deals on one hand with the phenomena which takes part in the formation of the heterogeneous spectrum of the pneumonias and, on the other hand, we will demonstrate the manner in which the expansion and the severity of the infectious phenomena depend on the virulence of the etiologic agent, on the manner in which the defence mechanisms have been overcome, and also on the biological state of the invaded organism.

Abbreviations: PN – pneumonia, O2 – oxygen, CO2 – carbon dioxide

The pulmonary ventilation ensures the maintenance of a pressure gradient between the alveolar air and the capillary blood so that the gaseous exchanges occur continuously. The atmosphere contains 78% nitrogen, 21% oxygen, 1% carbon dioxide, other gases, but also particles that comprise micro-flora. These particles have the potential to reach, together with the gases, an alveolar level contributing, from a phylogenetic point of view, to the development of certain defence mechanisms. Another issue is represented by the commune segments of the respiratory ways, which are usually colonized by bacteria and fungi, when a functional separation mechanism occurs and develops. 

 The "natural barriers" ensure the mechanical protection. The first natural barrier is the continuity and the integrity of the mucous membranes and of the respiratory epithelia. At the level of the nasal cavity, the vestibular nasal hair and the mucous secretion have the role of retaining the particles and the large sized fragments, which are completed with the sneezing reflex that has a role in the disposal of the already retained ones. The mucous, which is strongly vascularised, has a role in the warming and humidification of the air column and it strongly secrets mucous, having a mechanical role (it retains the particulates but also prevents the contact between the cellular receptors and adhesions or other adherence bacterial factors), and an immune role, through the lysozymes and opsonins (that is a heterogeneous group of substances which facilitates the contact between the cells, forming the immunity and the targets). 

 At the level of the pharynx and larynx an essential role is played by the mechanism for the automatic direction of the air and digestive way, which protects the airway from the risk of accidental entrance of food during the deglutition process (through the obstruction of the glottis by the epiglottis). The reflex, which is partially conditioned by coughing protects the airway, starting from the larynx, and prevents the accidental entrance of certain liquid or solid particles. At this level, the mucous is covered by a cylindrical monolayer epithelium – ciliated (the luminal pole of the epithelial cells presents approximately 100-200 cilia for each epithelial cell) which performs a curling coordinated movement in the aqueous layer of the mucous, performing the so-called mucous-ciliary clearance, which allows the transport of the impacted secretions and particulates up to the level of the glottis, from where they are expectorated or swollen. The ciliated epithelium is present up to the level of the respiratory bronchioles. The particulates which are less than 50 microns in size, solid or liquid, which do not have an impact up to this level, might reach together with the air column, to the alveolar level. 

 The immune mechanisms include cellular and humoral responses, non-specific and specific. 

 At the level of the sub-mucous there are immune-competent cells such as: tissue macrophage (histioblasts, dendritic cells, etc.) and circulating cells (monocytes), lymphocytes (B, T and NK), mastocytes, phagocytosis neutrophils leucocytes (microphage) but also basophiles and eosinophiles [**1**], which have a double role of intercepting any non-self elements which have managed to pass over the mechanical barriers, and cancelling them by means of the phagocytosis [**11**]. The process consists of several stages, which are identified through micro-cinematography [**12**]. 

 Upon the chemotactic action of certain local factors (from cellular death, the activation of the complement, antigens, etc.) the circulating cells pass through diapedesis from the capillary in the interstitial space, make their way to the target, issuing fixing frontal pseudopods accompanied by the traction of the rest of the cell on the new position, the contact with the target, either passive, hazardous, or active, with the intervention of certain facilitating factors (adhesins, specific or non-specific opsonins) and the phagocytosis, accompanied by the formation of a phagosome, which subsequently unites with a lysosome, resulting a phago-lysosome in which there is an attempt to perform the lisa of the particulate upon the action of the lysosomal enzymes. 

 At the level of the mucous, the activated B-lymphocytes locally release immunoglobulin, especially Ig. A secretor – but also Ig. M or even Ig. G, which have a role in the installation of immunity at the entrance gate on a non-determined period. There is a possibility that Ig. E involved in the activation and maintenance of respiratory sensitivity states and allergy episodes might occur. 

 In the absence of the ciliated epithelium, at the level of the terminal bronchioles, of the alveolar ducts and alveolus, the defence role is primarily played by the macrophages follower, by the lymphocytes and the polynuclears. Two main groups of macrophages were described – a group which is situated at the level of the superior airways and a second group, which are more numerous, which belong to the inferior airways and alveolus. Although they cannot be differentiated from a morphological point of view, the alveolus ones have more complex functional capacities as compared to the ones belonging to the inferior airways. They are the main alveolus cells that maintain their mobility and are capable to induce the phagocytosis – especially the one that is mediated by the opsonins. In non-pathogen conditions, the main function of these macrophages is represented by the clearance of the bacteria and of the inhaled particulates and the surfactant; they have the ability to suppress the activation of certain undesired inflammatory reactions and of a non-necessary immune response, but also to activate a defence reaction if the challenge is bigger. This mechanism includes the intervention of the cytokines and chemokines, which activate the defence reactions and of the eicosanoides, which also recruit in an inflammatory reaction the epithelia, the endothelia and the smooth muscles of the trachea-bronchial tract. In the end, the macrophages release Il.10 with a frenator role that induces the decrease of the local inflammation and the restoration of the normal functions of the airways and of the alveolus [**15**]. 

 The activation of the macrophages is dependent on the cytokines (TNF alpha), on the IFN.gamma, and on the D.3 hydroxi – vitamin. After the phagocytosis, the germs are killed by means of a series of cellular proteolysis enzymes and of active oxygen release. They are also able to partially degrade the phagocytized material with the maintenance of epitopes that they will send to the LT lymphocytes (CD.4 and CD.8) under the restriction of the major anti-genes of membrane hysto-compatibility (MHC.I and MHC.II). Their bactericide capacity might increase under the influence of certain T. helper lymphocytes or NK cells [**2**]. 

 The macrophages are not only activation cells but they also recruit other cells by releasing other molecules which stimulate the NK cells (TNF alpha), some T helper lymphocytes 1 and 2 (IL. 12 and IL.4) and they have a chemotactic effect for the polynuclear cells – through IL.1, IL.6, IL.8 and IL.B.4. 

 In addition, the pulmonary macrophages contribute both to the synthesis as well as to the degradation of the tissue matrix (through the secretion of some stimulating growing factors and of some cytokines which stimulate the cells which form the matrix, respectively through the secretion of certain metalloproteases which degrade the matrix and their inhibitors [**15**]. 

 The neutrophil polymorphonuclear leukocytes (PMN) migrate from the circulation within the sub-mucous (where their number is lower than the macrophages) under the influence of some selectines, integrins and of some derivates from Ig. represent the first group which is present at the location of the infection, in general; however, they are less important at the pulmonary level in the first stages of the defence reaction. Through the increased capacity of secreting the oxygen-free radicals, they collaborate with the macrophages to destroy those germs that are resistant to the isolated action of the macrophages. 

 The dendritic cell is the main cell that presents antigen; it has a medullar origin but on an independent line as compared to the T-lymphocytes and it is present both in the Waldeyer lymphatic formations as well as in the free dispersion at the level of the mucous. It presents characteristic cytoplasm prolongations that facilitate its capture of antigens and, although it has a lower phagic activity (lysosomal) as compared to the macrophages, it has a bigger capacity to transmit the antigenic epitopes due to the increased expression of the MHC.II molecules on their surface. After the capture of the antigen, it changes its shape and adherence and starts its lymphatic migration; in the ganglions, it recovers its shape once the captured antigen is presented to the T4 and T8 lymphocytes. It has a very short lifecycle (2-3 days) in the respiratory sub-mucous, but in the lymphatic formations it may survive for a longer period of time cooperating with LT. 

 The Natural Killer cell (NK) originates in the lymphocyte line without passing through the thymus; its development is dependent on the alpha and beta IFN, of the IL.2 and IL.15. It secretes alpha IFN, alpha TNF, IL.3 and GM-CSF; it acts in the early stages of the immune response, and it does not need previous contacts with the target cell or clone expansion (the case of the T and B-lymphocytes). 

 The non-specific humoral immunity is represented at respiratory level by a series of substances that have a defence role without the need to have previous conditions of identification of the non-self elements. This is the case of the lysozyme (already presented), which is present in the mucous secretions, and in the complement sub-mucous and – in a lower proportion, of the substances that have a chemo-tactic or opsonizant role; these substances might occur during the development of the defence mechanisms against certain germs. The defence against viruses also benefits from the presence of some interferons. 

 The complement is an enzyme complex with 11 components, defined with numbers from 1 to 9, in Yalle, in 1966; they progressively enter in a "cascade" activation process; during the activation, certain sub-components occur, which have defence helping roles. The cascade has the following development: 

 On a classical route through the antigen-antibody complexes, it activates the components in a series of reactions, finalised through the occurrence of the C.5-9 terminal lytic complex (called Membranary Attack Complex – MAC). This is the product which acts upon the target cell’s membrane and which perforates the membrane (the doughnut effect), which is sufficient to make the cell suffer from the destroying action of the water osmotic invasion and from the uncontrollable loss of its own constituents (ATP, ions) with a final lisis [**4**]. The classical route can also be activated in septic aggression conditions and due to the activated XII Factor (Hageman) and the reactive C protein attached to the bacterial membranes [**8**]. 

 The alternative or the properdin route comprises the short-circuited activation directly from the C.3 upon the action of non-specific tissue compounds (properdin) or bacterial (lipo-polysaccharides). The properdin seems to be a complex of protein substances which are part of the 1 and 2 groups of the alpha-globulins, and which take part at several immunologic processes – it facilitates the phagocytising of foreign cells or particles and during the inflammatory reactions. It is activated together with its attachment to the bacterial membranes, by replacing the antigen-antibody system in a more rapid activation of the complex. 

 The alternative route may also be activated, in certain conditions, by the plasmine, by the bacterial endotoxins and by certain polysaccharides of the bacterial wall [**8**].

**Table 1:**
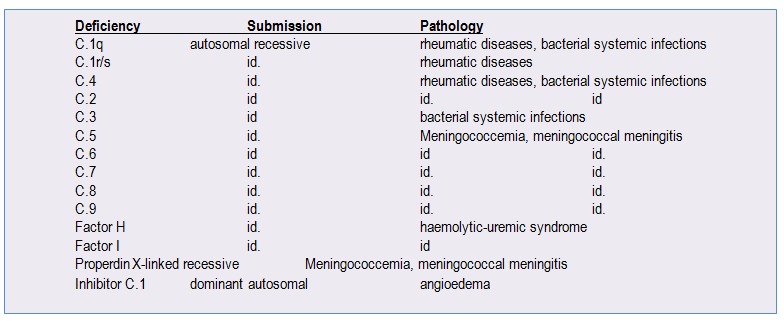
Congenital deficiencies of the Complement and the induced pathology [**13**]

 The interferon was discovered by Isaaks and Lindemann, the name being derived from the fact that it would interfere with some cellular defence processes. At present, it is known that the interferon is a protein substance, synthesised by different cells (especially by the ones involved in the defence processes such as the T-lymphocytes – especially the NK and K lines, the fibroblasts and the leucocytes) and afterwards extra-cellular released inducing the recruiting and the activation of other cells and processes, which have an immune – modulating effect. Several types of interferon are known (alpha, beta and gamma) which have different actions.

 The synthesis and the release of interferon increases under fever conditions and in ageing persons.

 It seems that the interferon would play a defence role not only in viral infections but also in some infections with Chlamydia and with protozoa (the Gondi toxoplasmosis, plasmodium bergei).

 The surfactant is a phospholipid complex (85%) -lipid (5%)-protidic (10%), which has a primordial role in the decrease of the superficial pressure at the level of the alveolus epithelia, contributing in an essential way to the maintenance of the alveolus and of the open terminal channels in both phases of the ventilation. Among other connected roles it also seems that it has a defence role through its A and D proteins. 

 So, Surfactant Protein A (SP-A) links to a wide range of micro-organisms and activates their elimination by the phagocyte cells, and SP.D, which has a structure that resembles the structure of the A protein, links to certain bacteria ( klebsiella pneumoniae, E. coli and pseudomonas aeruginosa), the defence capacity being increased for the aggregated form, which is that of oligomers [**9**].

 – The specific immunity is also ensured at the level of the respiratory apparatus by the same micro-organisms that are present in the entire organism. Every time the risk of penetration, overcome of the barriers and non-specific defence mechanisms occur, the more elaborated and more efficient specific, cellular and humoral mechanisms promptly interfere. 

 The main cells involved in these processes are the following:

 ∎ the cells which present antigen (monocytes, macrophages, dendritic cells)

 ∎ the T-lymphocytes (with the specialised sub-classes CD.4, CD.8 and others – Table 2)

 ∎ the B-lymphocytes 

 After a first contact with an intruding non-self element, which was captured and phagocytized by one of the specialised cells (ex. macrophage), a certain component which is antigenic representative for that particular element will be transmitted to some T-lymphocytes. The transfer process is conditioned by the existence and the acknowledgement of the molecules belonging to the major complex of HLA hysto-compatibility – type MHC class II for the T helper cells and MHC class I for the citotoxic T cells [**14**]. After the information is received, these cells will suffer a wide range of transformations – they become activated from an immunological point of view, being capable of releasing certain active substances in the extra-cellular environment (limphokines) which have different roles – activation and stimulation of the cells (LT.4 case – or helper), suppression of other types of cells (T.8 case – or suppression cells), cooperation (with the B-lymphocytes, with the macrophages) or with an opsonisant, chemotactic and toxic role for some of the target cells (the case of the lympho-toxins), pro-inflammatory role ("cutaneous inflammatory factor" and „the factor for the increase of the vascular permeability") or with an antiviral activity (interferon-like or even interferon) [**6**].

**Table 2:**
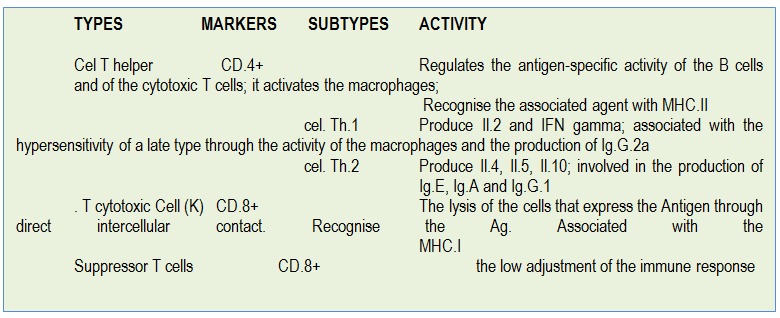
Subsets of T-lymphocytes (according to [**7**])

 The process is completed with the activation of the LB cells, which are the cells that produce immunoglobulin. The anti-genetic information processed by the macrophage will reach directly or through the CD.4 activated lymphocytes to the B-lymphocytes which will suffer in the same way, a process of differentiation and activation (becoming plasmocytes); they are extra-cellular immunoglobulin secretive capable of recognising the antigens. Schematically, the immunoglobulin possesses two sites which are active from an immunological point of view, at the edges of the molecule: a receptive situs for the antigen (called F.ab.) – on which it will be fixed‚ drawing in this manner the attention of the cells or of other mechanisms which are incapable of recognising the foreign antigen. The opposite molecular situs (called F.c.) will bond with this efector element. Therefore, by means of the immunoglobulin, the foreign antigenic particles will be recognised, marked and provided to the effectors in order for them to be annihilated. 

 The type M Immunoglobulin cells are the first to be formed and the most active (big molecules – the sedimentation constant is 19s, they do not have the capacity of crossing through biological membranes, but they are very greedy to get Ag; they are endowed with 5 receptors for each molecule). They are very efficient in the defence against foreign cells of large dimensions but they are less adequate for the small, monomolecular antigens, such as the endotoxins of the Gram-negative bacilli. Other disadvantages are the rapid catabolism and the relatively short circulatory life.

 They will be replaced by the type G Immunoglobulin cells, which have a smaller molecule (the sedimentation constant is 7s, only one receptor for Ag.) but which penetrates much better through the biological membranes; they are more efficient in the defence mechanism against the small sized antigens and have a long life cycle in the organism.

 The immunoglobulin type A secretor, dimmers of Ig.G have been described at the level of the respiratory and digestive sub-mucous also, but they lack the capacity to fix the complement and they do not posses antibodies – type activity (there are also Ig. A circulatory with unknown roles so far). It seems that these types of immunoglobulin play a more important role in the sensitisation process [**3**].

 The essential quality of the immune specific defence process is the capacity to retain the antigenic information obtained for a long period of time, even for a lifetime, through the differentiation of some clone cells, which retain memory (LT or LB) which ensure the prompt activation of the defence mechanisms immediately after the antigen has reappeared and was identified in the organism (the Booster phenomena). Through the accumulation of these infectious experiences solved in time, an adult organism would posses an immune heritage of approximately 10×12 lymphocytes and 10×18 specific antibodies [**4**].

 The general defence mechanisms in the respiratory infections are extremely complex. In this category, both certain local phenomena, such as the inflammation and the cicatrisation, the haemostasis and others, as well as some general reactions such as the febrile reaction, haemostasis (acid-basic, glycaemic, circulatory, etc.) and even the post – aggressive defence reaction (Selye) – which play the supreme neural-endocrine coordinating role, can be included.

 – Inflammation 

 It is a non-specific reaction of local defence, but also – in case of a prolonged or intense aggression – general (the systemic inflammatory response syndrome SIRS), it is a complex reaction through the wide range of locally and generally involved factors.

 In a classical approach, three stages of local inflammation can be described: the activation stage, the effecting stage and the healing stage. These stages can be found in the localised inflammatory processes, meaning in the septic aggressions (or aseptic aggression) of smaller sizes.

 The activation stage – in this situation, the direct action of the pathogen factors upon the tissues occurs immediately but also the indirect action occurs under the action of some cellular components locally released from the aggressed cells (proteolytic and hydrolytic enzymes and lysosomal proteolytic and hydrolytic). Biologically active substances occur; these substances have chemato-tactic, permeabilisation, activation of coagulation reactions effects of the kinin system, of the Complement, etc. The occurrence of these reaction elements will have as an effect the rapid transition to the second stage [**5**].

 The effector stage develops during the mobilisation and the amplification of the local reactions under the action of the mediators released locally. These mediators have diverse origins and are in an inactive state, that of pro-factors (protein, products for the degradation of the arachidonic acid, catecholamine), and they are also known as "reacting agents for the acute stage" (see Table 3). These mediators will induce vascular reactions (vasodilatation and the enhance of the local perfusion with the opening of the potential arterial-venous by-pass) and endothelial hyper-permeabilisation with serous exudation (including an increased input of energetic support, oxygen and defence factors) and chemotactic actions with the attraction of the reactive cells (macrophage, leucocytes, lymphocytes, etc). At local level, the classic signs described by Celsus are installed: rubor, tumor, calor, dolor, functio laesa.

**Table 3:**
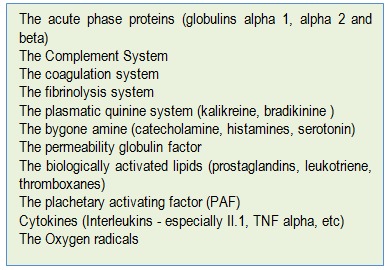
The acute phase reactants (well known)

 The cytokines are active polypeptides released from the cells involved in the defence reactions (macrophage, lymphocyte, granulocyte, endothelia) with a short life cycle. Their rapid disappearance from the system is one of the most efficient factors for the self-control of the inflammatory process. The most well known cytokines are Interleukin 1, which, together with the cachectin (TNF alpha) activate a wide series of adapting defence processes at the level of the entire organism [**10**].

 ∎ They induce fever through their action upon the hypothalamic centres 

 ∎ They induce leukocytosis with neotrophylia (and the deviation to the left of the Arneth formula) through action on the haematogenous marrow 

 ∎ They stimulate the phagocytosis through the direct action on the circulatory neutrophils 

 ∎ They stimulate the formation and the sedimentation of collagen through their action upon the fibroblasts 

 ∎ They induce metabolic acidosis through their action upon the smooth muscles 

 ∎ They induce the release from the liver of the acute phase proteins (the alpha, beta globulins, the reactive C protein, etc)

 ∎ They stimulate the release of the T and B-lymphocytes, of the limphokines and immunoglobulin which act as antibodies 

 Other cytokines recognised for being involved in the inflammatory processes are the following [**7**]:

 Interleukin 2 ∎ releases T and NK activated lymphocytes. Being known as the T-cell Growth Factor’ TCGF, it induces the proliferation of the T cells, the growth and the differentiation of the B cells, it activates the cyto-toxicity of the K and NK cells, it stimulates the secretion of I.l.4 and gamma-IFN. 

 Interleukin 3 – or the Colony Stimulating Factor (CSF), produced by the T and D-lymphocyte and by the mastocyt, – stimulates the proliferation of the stem cells from the haematogenous marrow but other lymphocytes also induce the growth of the mastocytes. 

 Interleukin 4 – or the B-Cell Growth factor (BCGF), is released by the T-lymphocytes and stimulates the proliferation of the B-lymphocytes and the secretion of Ig. G.1, Ig.A and Ig.G4 but also provides toxicity to the macrophages and stimulates the proliferation of the mastocytes. It also induces the activation and the growth of the T cells and the generation of LTC and interferes with the Th. switch in Th.2.

 Interleukin 5 ∎ or BCGF.2, released by the T cells which are stimulated and macrophages is not so well known in humans; in mice it proliferates the B-lymphocytes, makes differentiations between the eozinophils and stimulates the secretion of immunoglobulin G, M and A, erythropoietin and the acute phase proteins 

 Interleukin 6 – it occurs under the action of Il.1from the T and B lymphocytes, activated macrophages, monocytes, mastocytes, fibroblasts and hepatocytes; it stimulates the proliferation of the stem cells (as Il.3). It contributes to the formation of the acute phase proteins and of the antibodies from the B-lymphocytes, activates the cytotoxic lymphocytes K and NK, interacts synergically with I.l. 2 in the production of acute phase proteins in the hepatocytes and it also interacts with I.l.3 in the growth of the haematopoiesis, in the antiviral activity and in the neurons trophicity.

 Interleukin 7 – is located only in the haematogenous marrow where it is released (exactly as it is released from the tymic and splenic cells) and where it stimulates the young lymphocytes differentiation pre-B, and the immature tymocites. It stimulates the I.l.2 synthesis.

 Interleukin 8 (chemokine) is released from the monocytes, endothelia, alveolar macrophages, fibroblasts and T lymphocytes – It is chemotactic and induces the activation of the neutrophiles and of the T cells, it stimulates the basophiles’ chemotactism and the release of histamine; it also increases the vascular permeability.

 Interleukin 9 is released by the T lymphocytes and induces the proliferation of the T cells, the increase of the mastocytes and of the fever. 

 Interleukin 10 – is produced by the Th.2 lymphocytes, by the B activated cells, by the macrophages, and by the timocytes; it activates the B cells and the antibody-genesis, inhibits the function of the macrophages and the activation of MAC, interferes in the Th switch in Th.2, and has a chemotactic action for the Tc lymphocytes. It stimulates the antiviral defence.

 Interleukin 11, it is released from the fibroblasts of the bone marrow and stimulates the production of antibodies and of acute phase proteins, in synergy with I.l.3; it stimulates the ageing of the trombocytes.

 Interleukin 12 is formed in the cells which present antigen characteristics – macrophages, B-lymphocytes and Langerhans cells. It activates the T citotoxic cells (K) and NK, interferes with the Th switch in Th.2, and ensures protection towards the intracellular germs.

 Interleukin 15 occurs in monocytes, macrophages, epithelia and striated muscular cells. It induces the growth, the differentiation and the NK cytotoxicity; it stimulates the proliferation of the activated B cells. It is a co-stimulator of the synthesis of Ig.M and Ig.A, and stimulates the proliferation of the T cells and the synthesis of I.l.5.

 TNF. alpha – presented above, is released from the macrophages and monocytes and induces the occurrence of I.l.1, the activation of the neutrophils; it also induces the inflammatory process and the fever; it has cytotoxic and even cytostatic effects. 

 TNF.beta – or the Limphotoxin - is a cytokine released from the T-lymphocytes which has a cu cytotoxic action, destructive, against the target cells; it inhibits the growth of the T cells and the activation of the macrophages. 

 The stimulating factors of the colonies (GM-CSF, G-CSF, M-CSF) induce the increase of the granulocytes and monocytes. 

 TGF.alpha released from solid tumours and monocytes, and 

 TGF.beta released from trombocytes, placenta, bones, T and B cells. They both induce angiogenesis, bone resorption, the growth of tumours, the proliferation of collagen, the synthesis of collagen and fibronectin; it inhibits the NK and the proliferation of the B and T lymphocytes 

 The healing stage interpenetrates with the previous stage and will completely replace it. The cellular – proliferating processes with a reparatory purpose will now play a major role.

 The fibroblasts – which have migrated together with the other defence cells - will recondition the collagen matrix in parallel with the withdrawal of the exudative phenomena and with the proliferation – on this matrix, of the cells which are adequate for the interested tissue (the integrity of the endothelium, of the biological membranes, of the "noble" structures is reconstructed). If the destructions were too severe, the conjunctive, scar reparatory phenomena will prevail, in the detriment of the own cells. 

 The inflammatory agents are non-specific. They can have an infectious nature (bacteria, fungi, protozoa, viruses) and they can be physical agents (traumatisms, extreme temperatures that lead to the damage of the tissues, ionising radiations, penetrated foreign bodies, etc.) or chemical agents. They can also be exogenous (acids, bases, medicines) or endogenous (this is the case of the proteolysis enzymes upon the digestive epithelia in the acute pancreatitis, of the cellular constitutive from the cells destroyed in the inflammatory focal point, etc.)

 The persistence in the organism of an inflammatory agent in certain conditions – isolation, insularism, at the shelter of the defence mechanisms, or in the presence of some weak and inefficient defence mechanisms – might lead to the occurrence of chronic inflammatory phenomena, torpid, which are exposed to the risk of generating remote complication (septic, toxic, sensitivity, etc.).

 The febrile reaction

 It is a process usually associated with the inflammatory process, irrespective of its nature – either infectious or not (see Table 4). The fever mechanism consists of the dysfunctionality of the thermal homeostasis upon the direct and indirect action of the inflammatory cytokines (which are usually called "pyrogenic factors").

 The centre of the thermal regulation is situated in the di-encephala, and has an anterior hypothalamic nucleus, in the sympathetic area, which controls the thermogenesis mechanisms and a posterior hypothalamic nuclei, parasympathetic, which controls the thermolysis process.

 The thermogenesis involves the release of heat from the metabolic process, which takes place at the level of the liver (which has a normal temperature of 38–38.5 degrees C) and of the skeletal muscles (own temperature of 38 degrees Celsius) both in a repose state – through the permanent tonus – as well as in the contractile activity (including the shivers). The thermogenesis is performed by means of the endocrine performers (the thyroid hormones, the catecholamine and the glucocorticoids) and nervous (the pyramidal and extra-pyramidal system). The heat will be dispersed in the rest of the organism through the blood circulation.

 The thermolysis – or the loss of heat – is performed through the well-known physical processes of irradiation, convection and conduction and – additionally, through perspiration and its evaporation. 

 The two nuclei are in a permanent dynamic equilibrium, even cybernetic equilibrium, of mutual compensation, function of the signals received from the temperature receptors that are distributed into the entire body and of the local influence exercised by the temperature of the blood that irrigates the hypothalamus. This makes any abatement tendency from the normal temperature (diminished or added) to be immediately compensated through the intervention in the opposite direction of the complementary centre. For example, an exaggerated loss of temperature in cold conditions will promptly activate additional thermogenesis processes, and a tendency of loosing temperature will intensify the thermolysis processes. In this manner, the organism will be permanently adapted to the variable environmental conditions. 

**Table 4:**
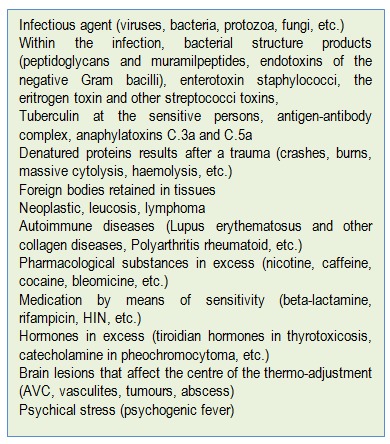
The activating factors of the febrile reaction (according to [**8**])

 Fever occurs only if, due to the direct action of the above mentioned pyrogenic factors, "the closing at a different level" of the equilibrium between the two nuclei occurs. 

 Pyrogenic exogenous and pyrogenic endogens were described.

 The exogenous pyrogens included all the factors – part of them being described in the table, which induce the fever. They act indirectly, through the release of the pyrogenic endogens. 

 The endogenous pyrogens are mainly the cytokines that have already been discussed: Il. 1 alpha and Il.1 beta, Il.2, TNF alpha, TNF beta (limphotoxine), Il.6 and IFN alpha. Experimentally, other leukins, the leukaemia inhibitor factor LIF, the cilia CNTF neurotropic factor and the M oncostatin were discovered. The intimate mechanism by means of which these factors induce the fever seems to consist of a di-encephalic action from the brain blood, by the local release of prostaglandins especially PG.E.2. This explains the antithermal direct action of the aspirin – which inhibits the release of prostaglandins.

 The endogenous pyrogens usually have a limited lifetime, depending on the presence of the activating factors. 

 Fever is a defence factor through the inhibition of the bacterial multiplication and of the viral replication; it stimulates the phagocitary cells and the activation of the LT and LB, the interferon synthesis and creates rheological conditions, which are favourable to the local blood flow. Moreover, at non-nocive values, the fever becomes a clinic reliable ally representing one of the most important diagnosis and prognostic parameters during the evolution of an infection. 

 At very increased values or prolonged durations, the fever can become an additional negative factor (it can activate convulsions at children, confusing states at older persons, prolonged catabolic syndromes, and consumptive syndromes, etc.) and must be diminished by all means – either physically (wrappings) and chemically (anti thermal medication and antiphlogistic medication).

 The systemic inflammatory syndrome (SIRS)

 SIRS integrates all the modifications of the organism installed as a defence response at the penetration of a foreign agent (non self) or own agent which is not recognised as a self (in cases of perturbation of the expression of the histo-compatibility antigens HLA.1 and HLA.2) during certain accidents or autoimmune diseases. 

 Generally speaking, this syndrome unites the reactions of the entire body mobilised in this answer: circulatory, respiratory, metabolic, humoral, endocrine, etc. – reactions which place the organism in a special situation, adapted to some additional functioning conditions.

 Clinical and laboratory elements are encountered in the definition of the SIRS.

 From a clinical point of view, during the objective examination, the following might occur: fever, cold sensation, shivers, profuse perspiration, deadness, facial erythema and/or diffuse, incurvation, headache, asthenia, tachycardia, with or without systemic hypotension, polypnea / tachypnea.

 The laboratory highlights the existence of certain alterations of "acute stage": leukocytosis, the HSR acceleration, hyperfibrinogenemia, hyperglobulinemia alpha and beta, reactive C protein. 

 In the context in which the laboratory investigation identifies a certain infectious agent in the hemoculture – or clinic elements which witness its presence in the circulation (the case of the metastatic abscesses, of some circulating elements having a bacterial structure, etc.) – we can talk about SEPSIS. 

 The spontaneous evolution or under an ineffective treatment of the above mentioned clinic phenomena can lead to an aggravation, to the state of septic shock and in the end to the plury-organic systemic insufficiency syndrome – SDOM – (the former MSOF "multi-system and organ failure") and exitus.

 The adjustment of the immune response takes place in stages, from the interest of first line elements for the simplest reactions to the most complex reactions – mentioned above – in the case of severe aggressions or long time aggressions. 

 The genetic deficiencies might have very severe repercussions upon the defence capacity reaction (congenital immune deficiency diseases or syndromes – Table 5.

**Table 5:**
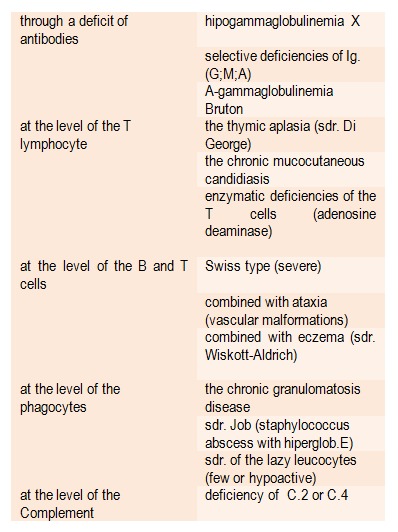
Syndromes of congenital immune deficiency (according to [**10**])

 It is essential that there are adjustment mechanisms for the adjustment of the intensity and of the amplitude of the defence reactions according to the aggression rate – not too much, not too less, not too short, not too long.

 The first recognised mechanism is an implicit self-control resulted from the cascade activation of the described stages, the short duration of the performers, the existence of some frenator factors (the T8 lymphocytes, antiproteases).

 In addition, the existence of a neuroendocrine "extra-control" is accepted and the release of the stimulating hormones for the basic functions (thyroidal, pancreatic, suprarenalian, hypophysis – the talamo-hypophysis-corticosuprarenalian axis).

 From the point of view of the infectious affection of the pulmonary parenchyma, besides the etiologic classification (bacterial, viral, parasites, fungi) or as radiological aspect (typical, atypical or mixed), the pneumonia can be a classified function of the entrance point (aerogenous, haematogenous), function of the epidemiologic condition (community-acquired / nosocomial), of the state of immuno-competency (pneumonia in the immune-depressed persons) or of the facilitating mechanisms (aspiration, etc.).

 a. The haematogenous pneumonia – are in reality broncho-pneumonia in isolated focal, multiple, disseminated, both lungs being interested usually. They are the quasi-mandatory expression of a septicaemia evolution in severe systemic bacterial infections with diverse entrance points, in the distance. The focal expresses as many septic micro-embolisations, each micro septic focal evolving on its own towards the restrained pneumonia blocks to the dimension of a lob or even less.

 b. The aspiration pneumonia represents a special group of aerogenous pneumonia particular through their etio-pathogenic condition. The common element to these pneumonia is their secondary development after the brutal penetration in the inferior airways of large sized foreign bodies or of the own oral-pharyngeal secretions.

 In the heterogeneous group of aspiration pneumonia, several clinic types can be distinguished 

 In case of aspiration of acid gastric secretion, a process of "chemical pneumonitis" is developed.. Clinically, besides the common elements (fever, tachypnea, cough), it presents bronchospasm, cyanosis and release of aerated sputa, and radiologically aired, they seem to be infiltrated in the delica segments. 

 The septic type is developed through the inhalation of the bucal flora and represents a typical model of bacterial pneumonia, with muco-purulent sputa and pulmonary infiltrated with a mixed aspect.

 After aspirating inert liquids (post immersion, for ex.), the initial picture indicates dyspnoea/apnoea, bronchospasm, cyanosis, inefficient cough, Radiological exam suggests pulmonary oedema. It can subsequently evolve towards the typical bacterial pneumonia.

 The inhalation of solid particles, depending on the dimensions, may lead to an acute picture of mechanical obstruction. The high obstruction leads to a suffocation image, accompanied even by death, and the distal obstructions lead to a subdued picture with frequent cough, chronic, irritative cough in the beginning and mucous-purulent afterwards, during the development of the secondary inflammatory process. 

 For these types of pneumonia, the particularity is also represented by the bacterial involved. It refers to the oropharyngeal flora – saprophyte or colonisation, – with the absence, usually, of the high pathogen germs but with the presence in more than 30% of the cases of the anaerobic germs (especially form the streptococcus group) of the staphylococcus, of the gram negative bacilli from the Haemophilus Influenzae group and of the fungi from the Candida group. Almost characteristic to the aspiration pneumonia, especially with anaerobe, is the election localisation either in the posterior segment of the superior right lobe, or in the superior segment of the inferior right lobe or in both of them, or in their corresponding left side.

 c. The community-acquired pneumonia 

 Definition. 

 The community-acquired pneumonias are defined functions of the epidemiologic and anamnestic context of the patient. In this category – the community-acquired category – that pneumonia which occurs in a non-hospitalised patient and with no recent or close contact (in the past 2 weeks) with a medical institution or of a continuous care (nursery homes, sanatoria, etc.) and without receiving any antibiotic treatments in the same previous period of time (irrespective of the pathology which needed that special treatment) is accepted. 

 d. The nosocomial pneumonia (PN)

 As definition, the pneumonia which occurs during a hospitalisation period – for other pathologies – for other pathologies – at more than 48 hours from the hospitalisation, or which will develop shortly after (maximum 2 weeks) the release from the hospital, practically as a continuation of the hospitalisation, are considered nosocomial. Through this epidemiologic condition, the nosocomial pneumonia are real infectious accidents on a compromised ground (at least partially) – through the existence of the other pathology which imposed the hospitalisation, and with the germs which are present in the hospitals, germs with a modified sensitivity to antibiotics through a selection pressure as a result of numerous treatments performed in the premises of that institution. 

 The pneumonia is nosocomial if: 

 ∎ It occurs after the first 72 hours after the hospitalisation;

 ∎ It is clinically objectivised through a condensation syndrome (and Rx. Through a pulmonary infiltration recently installed) and associates purulent expectoration, the isolation of the pathogen agent from the trans-tracheal aspirant, bronchi-alveolar lavage, blood or pulmonary biopsy;

 ∎ Specific antibodies occur in an increased titre;

 ∎ The hysto-pathological evidence for pneumonia is obtained. 

 The most severe form of PN was described in patients who have respiratory prostheses. The pneumonias installed in conditions of assisted ventilation are of four categories according to the moment of occurrence and to the presence of an antibiotic treatment in the past 15 days:

 ∎ Early PN occurred in the first 5 days of hospitalisation and in the absence of antibiotics 

 ∎ Early PN occurred in the first 5 days of hospitalisation but with prior antibiotic therapies 

 ∎ Tardive PN – after the fifth day, but with no prior antibiotic 

 ∎ Tardive PN with prior antibiotic

 e. Pneumonia in the immune-compromised patients 

 It occurs on the background of certain immune deficits, congenital or received (immune-depressed diseases or iatrogenic). They constitute clinically significant entities but not only through the frequently "exotic" aetiology (opportunistic flora or even saprophyte) but mostly because, through the mitigation of the immune and inflammatory responses, the clinical presence is modified profoundly, the imagistic aspects, mostly, the evolution, and the healing.

 In conclusion, the pneumonia constitutes a complex pathology realised through a combination of factors linked to the exceedance of the organism’s mechanisms of the defence by an etiologic agent.
